# Enhanced Passive Safety Surveillance (EPSS) confirms an optimal safety profile of the use of MF59^®^‐adjuvanted influenza vaccine in older adults: Results from three consecutive seasons

**DOI:** 10.1111/irv.12685

**Published:** 2019-10-16

**Authors:** Donatella Panatto, Mendel Haag, Piero Luigi Lai, Sylvie Tomczyk, Daniela Amicizia, Maria Maddalena Lino

**Affiliations:** ^1^ Department of Health Sciences University of Genoa Genoa Italy; ^2^ Interuniversity Research Center on Influenza and other Transmissible Infections (CIRI‐IT) Genoa Italy; ^3^ Clinical Development Seqirus Netherlands B.V. Amsterdam the Netherlands; ^4^ Seqirus USA Inc. Cambridge MA USA; ^5^ Seqirus IT SPA Siena Italy

**Keywords:** adjuvanted influenza vaccine, enhanced safety surveillance, Fluad™, influenza, pharmacovigilance; reactogenic

## Abstract

**Background:**

In Europe, the enhanced safety surveillance (ESS) of seasonal influenza vaccines is mandatory, in order to detect any potential increase in reactogenicity when the vaccine composition is updated. The MF59^®^‐adjuvanted influenza vaccine (Fluad™) is the first and the only licensed adjuvanted seasonal influenza vaccine in Europe.

**Objective:**

Our objective was to summarize the safety data of Fluad™ over three consecutive seasons.

**Methods:**

A passive approach to ESS (EPSS) was adopted, in which reporting of spontaneous adverse events (AEs) by vaccinees and vaccine exposure was estimated, in order to generate a near real‐time reporting rate. EPSS was conducted in Italy during the 2015, 2016, and 2017 influenza seasons in the primary care setting. All AEs reported within 7 days following immunization were analyzed by season, type and seriousness. Fisher's exact test was used to compare frequencies between seasons.

**Results:**

Total exposure accounted for approximately 1,000 doses of Fluad™ for each season. A total of 0.5% (2015), 0.7% (2016), and 0.5% (2017) individual case safety reports (ICSRs) were received, corresponding to a total of 9 (2015), 18 (2016), and 12 (2017) spontaneous AEs. The frequencies of AEs of interest were below those expected on the basis of the known safety profile of the vaccine. Most AEs were mild‐to‐moderate in severity. No between‐season difference was found.

**Conclusions:**

Our analyses confirmed that the safety data observed were consistent with the known safety profile of Fluad™, which has been amply established over the last 20 years. No significant changes in the safety profile were observed.

## 1 BACKGROUND

The prevention, monitoring, and control of adverse events (AEs) following immunization are essential to ensuring safety and maintaining public confidence in vaccines; without the latter it is almost impossible to achieve optimal vaccination coverage.^1^ Post‐marketing surveillance is crucial to ensuring the safety profile of a vaccine and identifying reports of unknown AEs. Compared with other vaccines, the pharmacovigilance activities for seasonal influenza vaccines have several distinctive characteristics. For example, the vaccine antigen composition may change twice a year (once for the northern hemisphere and once for the southern hemisphere), large population cohorts of different ages are immunized each year in a pre‐defined period of time, and the vaccine market is highly diversified according to national/regional immunization policies.^2^, ^3^, ^4^

In 2014, the European Medicines Agency (EMA) issued the document “Interim guidance on Enhanced Safety Surveillance (ESS) for seasonal influenza vaccines in the EU”.^3^ The aim of ESS is to promptly detect any clinically significant change in the frequency and/or severity of expected reactogenic events that can lead to a potentially more serious risk associated with influenza vaccination.^3^ Seqirus (formerly Vaccines Novartis Influenza) has implemented the EMA requirement and, starting from the 2015/2016 influenza season, carries out annual ESS of its products available in the EU, including the trivalent seasonal MF59^®^‐adjuvanted (Novartis International AG) influenza vaccine (Fluad™, Seqirus Inc).

Fluad™ was first licensed in 1997 in Italy; since then, 81 million doses have been administered worldwide.^5^ It is currently authorized for the immunization of people aged 65 years or older in about 30 countries, while in Canada a pediatric formulation indicated for children aged 6‐23 months is also available.^6^ Moreover, during the last 2009 pandemic, about 100 million doses of MF59^®^‐adjuvanted monovalent influenza vaccine were distributed and used in all principal target groups, including children from the age of 6 months and pregnant women.^5^

Basically, the MF59^®^ adjuvant is a squalene‐based oil‐in‐water emulsion. Squalene is a naturally occurring (also in humans) substance, being a direct pre‐cursor of cholesterol. The main purpose of including the MF59^®^ adjuvant is to enhance vaccine immunogenicity, which is particularly important in older adults, who are usually poorly responsive to traditional vaccines owing to immunosenescence.^5^, ^7^ Indeed, numerous^8^, ^9^, ^10^, ^11^, ^12^, ^13^ randomized controlled immunogenicity trials have shown that, compared with unadjuvanted formulations, Fluad™ induces significantly higher antibody titers against both homologous and heterologous virus strains, as well as higher seroconversion and seroprotection rates. Several cohort and case‐control studies^14^, ^15^, ^16^, ^17^, ^18^ have documented a higher in‐field effectiveness of Fluad™ vs unadjuvanted vaccines in reducing the number of laboratory‐confirmed influenza cases, hospitalizations for pneumonia and/or influenza, acute cardiovascular and cerebrovascular events and influenza‐like illness.^14^, ^15^, ^16^, ^17^, ^18^, ^19^

The safety and tolerability profile of Fluad™ is well‐established. An integrated analysis of 64 clinical trials^20^ revealed that people immunized with MF59^®^‐adjuvanted vaccines, in comparison with unadjuvanted vaccines, had a significantly lower risk of unsolicited AEs. Indeed, in the overall population, reports of any unsolicited AEs decreased by 35% [adjusted risk ratio, adjuvanted vs unadjuvanted: 0.65 (95% CI: 0.60‐0.70)]. With regard to solicited AEs, these were reported more frequently in subjects immunized with adjuvanted formulations than in those who received non‐adjuvanted vaccine. However, most of these AEs involved the injection site were of mild/moderate intensity and resolved in a few days.^20^ In a 3‐year prospective cohort study^21^ (88 449 doses of Fluad™ and 82 539 doses of unadjuvanted vaccines) the frequency of AEs of special interest (serious) resulting in hospitalization was very low and similar (not statistically significant) in the two vaccine groups. A systematic evaluation of pharmacovigilance reports (without inferring causality),^22^ covering a period of 9 years in which 27 million people were vaccinated with Fluad™, revealed that the reporting rate of serious AEs was very low and in line with the expected reporting frequency in the general population.

The primary objectives of the present report were to summarize the frequency of AEs reported by subjects aged ≥65 years within 7 days following immunization with Fluad™ in three consecutive influenza seasons within the context of ESS and to compare the observed frequencies of AEs with the existing safety data on Fluad™.

The secondary objective was to compare the observed frequencies between seasons.

## 2 METHODS

### 2.1 Enhanced passive safety surveillance (EPSS)

The Interim guidance document^3^ specifies three options for ESS: active surveillance, passive surveillance, and data mining/use of electronic health record data. As a part of its routine pharmacovigilance activities Seqirus implemented the EPSS. EPSS uses non‐interventional methods for the collection of exposure data, facilitating spontaneous AE reports, and near real‐time collection of the number of vaccinations. The EPSS approach facilitates and enhances the quality of spontaneous AE reporting by means of the following activities: (a) raising vaccinees' awareness of the importance of reporting AEs, (b) providing vaccinees with contact information for reporting AEs following vaccination, (c) providing vaccinees with uniquely coded “vaccination cards” containing detailed information on the vaccine administered, in order to facilitate the reporting/identification process, and (d) near real‐time collection of the number of vaccinations.

### 2.2 Surveillance design and setting

The EPSS protocols complied with the Interim guidelines.^3^ The study was non‐interventional, since the decision to utilize a given vaccine was a part of routine care and was left entirely to the discretion of general practitioners (GPs). Only non‐interventional methods were applied to data collection. Surveillance was conducted in Italy in a multi‐center setting (17‐19 sites each season) in three consecutive (2015/2016, 2016/2017, and 2017/2018) influenza seasons by the Inter‐University Centre for Research on Influenza and other Transmissible Infections (CIRI‐IT), Genoa (Italy). Until 2017/2018, the CIRI‐IT was a part of the Italian sentinel surveillance of influenza (InfluNet) and coordinated the activities of sentinel physicians in an area covering nine (out of 20) Italian regions and approximately 2% of the Italian population. Since 2017/2018, InfluNet has changed, but CIRI‐IT has continued its activity of influenza surveillance.

The surveillance activities were planned to include a total of 1000 routine exposures to Fluad™. According to the summary of product characteristics (SPC),^23^ Fluad™ should be administered to subjects aged ≥65 years.

Following routine vaccination, GPs and their staff instructed vaccinees to report any AE in general, and specifically those occurring in the first 7 days. A standardized and uniquely numbered vaccination card with specific information on the batch, brand, date of vaccine administration, and the contact number for reporting spontaneous AEs was issued to vaccinees. Vaccinees were also informed that they could report AEs either by phoning the call center or directly to the health authorities, as indicated on the vaccination card.

### 2.3 Data collection, review, and analysis

All spontaneous individual case safety reports (ICSRs) of AEs were collected and processed by the trained staff of a toll‐free call center through a structured interview. In particular, the following information was systematically gathered: description of the events experienced, chronology of events, severity, outcome as well as demographics, medical history, ongoing health conditions, and concomitant vaccination/medications. The reports included the vaccination card number to enable identification of the spontaneous AEs which originated from the same sample population which was the denominator of the surveillance. Multiple contacts with the call center for the same vaccination card number (either for reports of different AEs or of the same event reported by different reporters) were handled as a separate ICSR with its own unique reference number. The total number of Fluad™ doses administered was collected directly from GPs.

Reports of spontaneous AEs received at the call center were entered in an electronic safety report capture tool. All AEs were promptly translated and encoded by the call center staff using the medical dictionary for regulatory activities (MedDRA^®^) of the ongoing version at the time of the surveillance. Each report was assigned a unique reference number.

To be considered eligible spontaneous AEs had to be reported to the call center as a part of EPSS and to meet the following criteria of a valid report: ≥1 identifiable reporter, ≥1 identifiable vaccinee, ≥1 suspected adverse reaction, and ≥1 suspected medicinal product (suspected vaccine product). Moreover, ICSRs that occurred beyond 7 days following vaccination were out of scope of the EPSS (and therefore were not included in the current analysis) but were included in the continuous routine evaluation of safety data in the global safety database. All eligible AEs were reviewed on a weekly basis.

### 2.4 Data analysis

The primary outcome was the number of ICSRs per 100 doses administered, that is the ICSR reporting rate (%). The denominator corresponded to the total number of Fluad™ doses administered, and the latter data being collected directly from GPs. ICSRs with at least one serious AE were analyzed separately and reported as a proportion (%) of the total number of ICSRs. The reporting rate (%) of reactogenic AEs of interest (rAEIs) was then calculated, as recommended by the Interim guidelines.^3^ rAEIs are local, systemic, or allergic reactions that may indicate a potential for more serious risks associated with exposure to the vaccine. This report included the following rAEIs: fever (including high‐grade fever > 39°C), nausea, vomiting, malaise, headache, decreased appetite, myalgia, arthralgia, rash, events indicative of allergic and hypersensitivity reactions (including ocular symptoms) and injection site reactions (including pain, erythema, and swelling). rAEIs were also described according to seriousness (serious and non‐serious) and their observed reporting rates were compared with the expected rates as per the SPC of Fluad™.^24^ Subsequently, AEs constituting risk for risk management plan (RMP) were described and analyzed; the following AEs were considered: anaphylaxis, extensive limb swelling, convulsions, neuritis, encephalitis, vasculitis, Guillain‐Barré syndrome, demyelination disorders, Bell's palsy, immune thrombocytopenia, hemolytic disorders, and vaccination failure. Finally, other spontaneous AEs and the off‐label use of Fluad™ (ie, vaccine administration to subjects < 65 years) were also described.

All data were described separately for each influenza season. Data were reported in absolute and relative (%) numbers with 95% confidence intervals (CIs) computed by means of the exact method. Fisher's exact test was used to compare the reporting rates across seasons.

### 2.5 Ethics statement

The study was conducted in full accordance with good pharmacovigilance practices.^25^ As required, the study protocol was submitted annually to the Ethics Committee of the Liguria Region (Genoa, Italy) and was approved.

## 3 RESULTS

### 3.1 Overall exposure data

In the three seasons, the study was carried out mainly in the month of November (November 4, 2015‐November 28, 2015, November 2, 2016‐December 3, 2016, and November 6, 2017‐December 5, 2017, respectively). In all three seasons, the target exposure number was reached earlier than expected. Total exposure accounted for 1060, 1046, and 1045 doses of Fluad™ in the seasons 2015/2016, 2016/2017, and 2017/2018, respectively.

### 3.2 Analysis of individual case safety reports (ICSRs)

A total of 5 (0.5%; 95% CI: 0.2%‐1.1%), 7 (0.7%; 95% CI: 0.3%‐1.4%), and 5 (0.5%; 95% CI: 0.2%‐1.1%) ICSRs were received in seasons 2015/2016, 2016/2017, and 2017/2018, respectively. No between‐season variation in the reporting rate was observed (*P* = .83). The ICSRs analyzed contained a total of 9 (0.8%; 95% CI: 0.4%‐1.6%), 18 (1.7%; 95% CI: 1.0%‐2.7%), and 12 (1.1%; 95% CI: 0.6%‐2.0%) AEs (in relation to the total doses administered) in seasons 2015/2016, 2016/17, and 2017/2018, respectively, with no significant difference (*P* = .20) among seasons.

Cumulatively, over the three seasons, two ICSRs involved at least one AE that was assessed as serious. The first (season 2015/2016, 20.0% of total ICSRs) concerned an 85‐year‐old woman with back pain onset 4 days after a dose of Fluad™. Despite the temporal relationship, there was no sufficient evidence to link the back pain with Fluad™ administration. The second (season 2016/2017, 14.3% of total ICSRs) involved a 79‐year‐old woman who was hospitalized after presenting with headache, visual impairment, and cerebral hemorrhage 2 days after receiving Fluad™. This patient had a spine pathology and was taking lansoprazole and a fixed combination of paracetamol and codeine as concomitant medications. At the time of reporting, the outcome was not recorded. Despite the temporal relationship, there was insufficient supporting evidence linking influenza vaccination with the cerebral hemorrhage (diagnostic imaging or laboratory tests to exclude other potential etiologies). No serious AEs (0%) were reported in the 2017/2018 season.

### 3.3 Analysis of reactogenic adverse events of interest (rAEIs)

As shown in Table 1, a total of 22 rAEIs [3 (0.3%; 95% CI: 0.1%‐0.8%), 12 (1.1%; 95% CI: 0.6%‐2.0%), and 7 (0.7%; 95% CI: 0.3%‐1.4%) rAEIs in influenza seasons 2015/2016, 2016/2017, and 2017/2018, respectively] were reported, with non‐significant differences between seasons (*P* = .058). The observed frequency was lower than expected on the basis of the known safety profile of the product. A large overlap of the exact 95% CIs of the reporting rates of single rAEIs suggested no between‐season variation. Across the three seasons, only one rAEI was classified a serious: a case of headache reported in the 2016/2017 season (described above).

**Table 1:** Expected and observed reactogenic adverse events of interest (rAEIs), by season

rAEI	Expected frequency	Observed frequency, N (%; 95%CI)*		
		Influenza season		
		2015/2016	2016/2017	2017/2018
Fever (any)	≥1/100, <1/10	2 (0.2; 0.0‐0.7)	4 (0.4; 0.1‐1.0)	3 (0.3; 0.1‐0.8)
High fever (>39°C)	NA	0	0	0
Nausea	≥1/100, <1/10	0	0	0
Vomiting	≥1/100, <1/10	0	0	0
Malaise	≥1/100, <1/10	0	2 (0.2; 0.0‐0.7)	2 (0.2; 0.0‐0.7)
Headache	≥1/10	0	1 (0.1; 0.0‐0.5)	0
Decreased appetite	NA	0	0	0
Myalgia	≥1/10	0	0	0
Arthralgia	≥1/100, <1/10	0	0	0
Rash	≥1/1000, <1/100	0	0	0
Events indicative of allergic and hypersensitivity reactions, including ocular symptoms	NA	0	0	0
Injection site reactions (eg, pain, erythema, and swelling)	≥1/100, <1/10	1 (0.1; 0.0‐0.5)	5 (0.5; 0.2‐1.1)	2 (0.2; 0.0‐0.7)

* The exact upper limit of the 95% CI for zero reports is 0.4%.

### 3.4 Analysis of reports per adverse event defined as risk in the risk management plan (RMP)

Across the three seasons, one AE was identified as risk per RMP. This was a case of extensive limb swelling reported in the 2016/2017 season. Specifically, a 68‐year‐old vaccinee developed injection site swelling, pain, and erythema on the same day as vaccination with Fluad™. A topical heparinoid was applied, but the AEs at the time of the report were still not resolved. This ICSR contained the preferred term “injection site swelling”, which is used to identify potential cases of extensive limb swelling, an identified risk of Fluad™ according to the RMP. However, review of the narrative did not indicate any extensive or large swelling, or peripheral swelling crossing of the joints of the limb involved. Hence, identification of this risk is limited to the preferred term and is not clinically consistent with extensive limb swelling.

### 3.5 Analysis of other spontaneous adverse events and off‐label use

Other (not previously described) spontaneous AEs were generally single (N = 1) events and included increased glycemia, cough, dizziness, nasopharyngitis, and oropharyngeal pain in the 2015/2016 season, influenza‐like illness in the 2016/2017 season and chills, feeling hot and pollakiuria in the 2017/2018 season. Only the AEs “chills” (N = 2 in the 2017/2018 season) and “fatigue” (N = 2 in 2016/2017 and N = 1 in 2017/2018) accounted for more than one report and/or were observed in more than one season. In sum, all other AEs were classified as uncommon (≥1/1000, <1/100) and were reported with a lower frequency than expected on the basis of the SPC.

Off‐label use accounted for <0.5% in all three seasons, with no evidence (*P* = .73) of any between‐season difference [2015/16: N = 4, 0.4% (95% CI: 0.1%‐1.0%); 2016/17: N = 2, 0.2% (95% CI: 0.0%‐0.7%); 2017/18: N = 4, 0.4% (95% CI: 0.1%‐1.0%)]. No ICSR came from vaccinees aged <65 years.

## 4 DISCUSSION

This report is the first to provide an overview of spontaneous ICSRs received from vaccinees with Fluad™ in three consecutive influenza seasons in the context of EPSS. The observed overall safety profile of Fluad™ proved to be consistent with the currently known safety profile of the product. No clinically relevant safety information (due to unexpected spontaneous reporting frequency, intensity, or nature of events) that had not been previously identified through routine post‐marketing surveillance emerged. Only two ICSRs involved at least one AE that was assessed as serious. Despite the temporal relationship, there was no sufficient evidence to link these adverse events with Fluad™ administration.

Overall, EPSS may be a useful approach to support the early detection of possible safety changes in a near real‐time modality; this has been previously documented by EPSS of a trivalent split virion inactivated influenza vaccine (Vaxigrip™, Sanofi Pasteur) and an intradermal trivalent split virion inactivated 15 µg (Intanza™ 15 µg, Sanofi Pasteur) vaccine.^24^, ^25^ Our well‐established EPSS of Fluad™ will therefore continue in the next influenza season.

The present study has both strengths and limitations. A particular strength lies in the fact that the population was homogeneous, which enabled us to make a direct between‐season comparison. In all three seasons, EPSS was conducted in Italy by the same GPs, who monitored the same population in almost the same period (ie, close to the start of the national immunization campaign and before the peak of disease). Furthermore, vaccine recommendations remained unchanged during the study period. Indeed, it is known that AE reporting rates may differ both temporally^26^ and geographically.^26^, ^27^ Italy was chosen as a reference country since Fluad™ is one of the oldest brands available in this country and, at the time of the study, had a very high market share in subjects aged ≥65 years. This latter fact also enabled us to reach a target population size of 1000 doses relatively quickly and efficiently. Furthermore, rigorous quality control and the use of vaccination cards enabled us to identify/attribute data unambiguously, thus minimizing the risk of misclassification.

The study limitations are basically inherent in the nature of passive surveillance activities. First, the spontaneous reporting of AEs may result both in under‐reporting (whereby only a fraction of the total number of AEs occurring after vaccination are reported) and in differential reporting (whereby more serious AEs and those with a shorter onset time after vaccination are more likely to be reported during the surveillance period than minor AEs or those with a longer time to onset). Second, owing to the short‐term nature of the EPSS, it may be difficult to accurately estimate the rates of certain potential/identified risks per RMP that are rare, more complex to diagnose, of multifactorial etiology, and/or exhibit long latency.

We believe that these data are of primary importance to stakeholders in countries where Fluad™ is authorized. Moreover, considering the recent preferential recommendations on the use of Fluad™ in subjects aged ≥65 and ≥75 years issued by Public Health England^28^ and the Italian Ministry of Health,^29^ respectively, we may expect a significantly larger population to be vaccinated with Fluad™ in the upcoming influenza seasons. For instance, Fluad™ will be used preferentially in the UK in all over 65‐year olds^29^; this means that the number of doses administered is likely to rise from zero to approximately 9‐10 million (assuming a vaccination coverage of 75%^30^ among the 11.8 million people aged ≥65 years^31^). The present report will therefore be very useful in these jurisdictions.

To conclude, Fluad™ is the first, and currently the only, available seasonal influenza vaccine in Europe that has been specifically designed to overcome immunosenescence, a phenomenon affecting older adults. Our analyses confirmed that the safety data that emerged from passive ESS over three consecutive seasons were consistent with the known safety profile of Fluad™, which has been amply established over the last 20 years. Indeed, no significant changes in the safety profile of Fluad™ were observed.

## CONFLICT OF INTEREST

Mendel Haag, Sylvie Tomczyk, and Maria Maddalena Lino are full‐time employees of Seqirus, a CSL company, and hold shares in CSL ltd. Donatella Panatto, Piero Luigi Lai, and Daniela Amicizia declare no conflict of interest.

## References

[1] Zanoni G , Berra P , Lucchi I , et al. Vaccine adverse event monitoring systems across the European Union countries: time for unifying efforts. Vaccine. 2009;27:3376‐3384. 19200851 10.1016/j.vaccine.2009.01.059

[2] Moro PL , Li R , Haber P , Weintraub E , Cano M . Surveillance systems and methods for monitoring the post‐marketing safety of influenza vaccines at the centers for disease control and prevention. Expert Opin Drug Saf. 2016;15(9):1175‐1183. 27268157 10.1080/14740338.2016.1194823 PMC6500454

[3] European Medicines Agency (EMA) . Interim guidance on enhanced safety surveillance for seasonal influenza vaccines in the EU. http://www.ema.europa.eu/docs/en_GB/document_library/Scientific_guideline/2014/04/WC500165492.pdf. Accessed on 15 September, 2018.

[4] Treanor JJ . Clinical practice. Influenza vaccination. N Engl J Med. 2016;375(13):1261‐1268. 27682035 10.1056/NEJMcp1512870

[5] O'Hagan DT , Rappuoli R , De Gregorio E , Tsai T , Del Giudice G . MF59 adjuvant: the best insurance against influenza strain diversity. Expert Rev Vaccines. 2011;10(4):447‐462. 21506643 10.1586/erv.11.23

[6] Canadian Paediatric Society (CPS) . Vaccine recommendations for children and youth for the 2017/2018 influenza season. https://www.cps.ca/en/documents/position/influenza-vaccine-recommendations. Accessed on 15 October, 2018.

[7] Martin JT . Development of an adjuvant to enhance the immune response to influenza vaccine in the elderly. Biologicals. 1997;25(2):209‐213. 9236054 10.1006/biol.1997.0086

[8] Banzhoff A , Nacci P , Podda A . A new MF59‐adjuvanted influenza vaccine enhances the immune response in the elderly with chronic diseases: results from an immunogenicity meta‐analysis. Gerontology. 2003;49(3):177‐184. 12679609 10.1159/000069172

[9] Beyer WE , Nauta JJ , Palache AM , Giezeman KM , Osterhaus AD . Immunogenicity and safety of inactivated influenza vaccines in primed populations: a systematic literature review and meta‐analysis. Vaccine. 2011;29(34):5785‐5792. 21624411 10.1016/j.vaccine.2011.05.040

[10] Frey SE , Reyes MR , Reynales H , et al. Comparison of the safety and immunogenicity of an MF59®‐adjuvanted with a non‐adjuvanted seasonal influenza vaccine in elderly subjects. Vaccine. 2014;32(39):5027‐5034. 25045825 10.1016/j.vaccine.2014.07.013

[11] Gasparini R , Pozzi T , Montomoli E , et al. Increased immunogenicity of the MF59‐adjuvanted influenza vaccine compared to a conventional subunit vaccine in elderly subjects. Eur J Epidemiol. 2001;17(2):135‐140. 11599686 10.1023/a:1017919305501

[12] Li R , Fang H , Li Y , Liu Y , Pellegrini M , Podda A . Safety and immunogenicity of an MF59‐adjuvanted subunit influenza vaccine in elderly Chinese subjects. Immun Ageing. 2008;5:2. 18289372 10.1186/1742-4933-5-2 PMC2291031

[13] Ansaldi F , Zancolli M , Durando P , et al. Antibody response against heterogeneous circulating influenza virus strains elicited by MF59‐ and non‐adjuvanted vaccines during seasons with good or partial matching between vaccine strain and clinical isolates. Vaccine. 2010;28(25):4123‐4129. 20433807 10.1016/j.vaccine.2010.04.030

[14] Van Buynder PG , Konrad S , Van Buynder JL , et al. The comparative effectiveness of adjuvanted and unadjuvanted trivalent inactivated influenza vaccine (TIV) in the elderly. Vaccine. 2013;31(51):6122‐6128. 23933368 10.1016/j.vaccine.2013.07.059

[15] Mannino S , Villa M , Apolone G , et al. Effectiveness of adjuvanted influenza vaccination in elderly subjects in northern Italy. Am J Epidemiol. 2012;176(6):527‐533. 22940713 10.1093/aje/kws313 PMC3447603

[16] Spadea A , Unim B , Colamesta V , et al. Is the adjuvanted influenza vaccine more effective than the trivalent inactivated vaccine in the elderly population? Results of a case–control study. Vaccine. 2014;32(41):5290‐5294. 25087677 10.1016/j.vaccine.2014.07.077

[17] Puig‐Barberà J , Díez‐Domingo J , Varea AB , et al. Effectiveness of MF59‐adjuvanted subunit influenza vaccine in preventing hospitalisations for cardiovascular disease, cerebrovascular disease and pneumonia in the elderly. Vaccine. 2007;25(42):7313‐7321. 17889411 10.1016/j.vaccine.2007.08.039

[18] Iob A , Brianti G , Zamparo E , Gallo T . Evidence of increased clinical protection of an MF59‐adjuvant influenza vaccine compared to a non‐adjuvant vaccine among elderly residents of long‐term care facilities in Italy. Epidemiol Infect. 2005;133(4):687‐693. 16050515 10.1017/s0950268805003936 PMC2870297

[19] Domnich A , Arata L , Amicizia D , Puig‐Barberà J , Gasparini R , Panatto D . Effectiveness of MF59‐adjuvanted seasonal influenza vaccine in the elderly: A systematic review and meta‐analysis. Vaccine. 2017;35(4):513‐520. 28024956 10.1016/j.vaccine.2016.12.011

[20] Pellegrini M , Nicolay U , Lindert K , Groth N , Della CG . MF59‐adjuvanted versus non‐adjuvanted influenza vaccines: integrated analysis from a large safety database. Vaccine. 2009;27(49):6959‐6965. 19751689 10.1016/j.vaccine.2009.08.101

[21] Villa M , Black S , Groth N , et al. Safety of MF59‐adjuvanted influenza vaccination in the elderly: results of a comparative study of MF59‐adjuvanted vaccine versus nonadjuvanted influenza vaccine in northern Italy. Am J Epidemiol. 2013;178(7):1139‐1145. 23863759 10.1093/aje/kwt078 PMC3783089

[22] Schultze V , D'Agosto V , Wack A , Novicki D , Zorn J , Hennig R . Safety of MF59 adjuvant. Vaccine. 2008;26(26):3209‐3222. 18462843 10.1016/j.vaccine.2008.03.093

[23] Italian Medicine Agency . Fluad, summary of product characteristics. https://farmaci.agenziafarmaco.gov.it/aifa/servlet/PdfDownloadServlet?pdfFileName=footer_004166_031840_RCP.pdf%26retry=0%26sys=m0b1l3.Accessed on 5 September, 2018.

[24] Chabanon AL , Bricout H , Ballandras C , Souverain A , Caroe TD , Butler KM . Report from enhanced safety surveillance of two influenza vaccines (Vaxigrip and Intanza 15 µg) in two European countries during influenza season 2016/17 and comparison with 2015/16 season. Hum Vaccin Immunother. 2018;14(2):378‐385. 29148911 10.1080/21645515.2017.1405882 PMC5806654

[25] Bricout H , Chabanon AL , Souverain A , Sadorge C , Vesikari T , Caroe TD . Passive enhanced safety surveillance for Vaxigrip and Intanza 15 µg in the United Kingdom and Finland during the northern hemisphere influenza season 2015/16. Euro Surveill. 2017;22(18);30527. 28494843 10.2807/1560-7917.ES.2017.22.18.30527 PMC5434878

[26] Marrero O , Hung EY , Hauben M . Seasonal and geographic variation in adverse event reporting. Drugs Real World Outcomes. 2016;3(3):297‐306. 27747826 10.1007/s40801-016-0081-6 PMC5042937

[27] Joelson S , Joelson IB , Wallander MA . Geographical variation in adverse event reporting rates in clinical trials. Pharmacoepidemiol Drug Saf. 1997;6(Suppl 3):S31‐S35. 15073752 10.1002/(sici)1099-1557(199710)6:3+<s31::aid-pds288>3.3.co;2-w

[28] Public Health England (PHE) . Guidance. Summary of data to support the choice of influenza vaccination for adults in primary care. https://www.gov.uk/government/publications/flu-vaccination-supporting-data-for-adult-vaccines/summary-of-data-to-support-the-choice-of-influenza-vaccination-for-adults-in-primary-care. Accessed on 16 October, 2018.

[29] Italian Ministry of Health . Prevention and control of influenza: recommendations for the season 2018/19. http://www.trovanorme.salute.gov.it/norme/renderNormsanPdf?anno=2018%26codLeg=64381%26parte=1%2520%26serie=null. Accessed on 5 September, 2018.

[30] European Centre for Disease Prevention and Control (ECDC) . Seasonal influenza vaccination and antiviral use in Europe–Overview of vaccination recommendations and coverage rates in the EU Member States for the 2013–14 and 2014–15 influenza seasons. Stockholm, Sweden: ECDC; 2016.

[31] UK Office for National Statistics . Overview of the UK population: July 2017 https://www.ons.gov.uk/peoplepopulationandcommunity/populationandmigration/populationestimates/articles/overviewoftheukpopulation/july2017. Accessed on 5 September, 2018.

